# Supplementary material for: The Systems Biology Research Tool: evolvable open-source software
Source: BMC Syst Biol. 2008 Jun 29;2:55. doi: 10.1186/1752-0509-2-55 (PMC2446383; doi:10.1186/1752-0509-2-55)
Supplement: Additional file 1 — SBRT Archive. An archive of the current version of the Systems Biology Research Tool. [file 1752-0509-2-55-S1.zip › sbrt-1.4.0/doc/developers_guide/api/sbrt/shell/text/SimpleListFormat.html]

SimpleListFormat


|  |  |  |
| --- | --- | --- |
| **Overview** \| **Package** \| **Class** \| **Use** \| **Tree** \| **Deprecated** \| **Index** \| **Help** | |  |
| **PREV CLASS**   **NEXT CLASS** | **FRAMES**    **NO FRAMES**     **All Classes** |  |
| SUMMARY: NESTED \| FIELD \| CONSTR \| METHOD | DETAIL: FIELD \| CONSTR \| METHOD |  |


---


## sbrt.shell.text Class SimpleListFormat<E>

```
java.lang.Object
  sbrt.shell.text.SimpleListFormat<E>
```

**Type Parameters:**: `E` - the type of element.

**All Implemented Interfaces:**: Format, Formatter<java.util.List<E>>, Parser<java.util.List<E>>, SimpleFormat<java.util.List<E>>

---

``` public class SimpleListFormat<E> extends java.lang.Object implements SimpleFormat<java.util.List<E>> ```

This interface is used to wrap `ListFormat` objects as `SimpleFormat`
objects.

**Author:**
:   This interface was written and documented by
    Jeremiah Wright while in the Wagner lab.

---

**Constructor Summary**

`SimpleListFormat(ListFormat<E> listFormat)`
  
          Constructs a new simple list format from the provided list format.


| **Method Summary** | |
| --- | --- |
| `java.lang.String` | `format(java.util.List<E> list)`             Returns a formatted string representation of the provided list. |
| `Formatter<E>` | `getElementFormatter()`             Returns the formatter for the elements of this list format. |
| `Parser<E>` | `getElementParser()`             Returns the parser for the elements of this list format. |
| `ListFormat<E>` | `getListFormat()`             Returns the underlying list format. |
| `java.util.List<E>` | `parse(java.lang.String list)`             Parses the provided string and returns its corresponding collection of elements as a list. |

**Methods inherited from class java.lang.Object**

`clone, equals, finalize, getClass, hashCode, notify, notifyAll, toString, wait, wait, wait`

**Methods inherited from interface sbrt.shell.text.Formatter**

`format`

**Constructor Detail**

### SimpleListFormat

```
public SimpleListFormat(ListFormat<E> listFormat)
```

:   Constructs a new simple list format from the provided list format.

    **Parameters:**: `listFormat` - the list format to be wrapped.


**Method Detail**

### format

```
public java.lang.String format(java.util.List<E> list)
```

:   Returns a formatted string representation of the
    provided list.

    :   **Parameters:**: `list` - the list to be formatted. **Returns:**: a formatted string representation of the provided list.

---


### parse

```
public java.util.List<E> parse(java.lang.String list)
```

:   Parses the provided string and returns its
    corresponding collection of elements as a list.

    :   **Specified by:**: `parse` in interface `Parser<java.util.List<E>>`
    :   **Parameters:**: `list` - the string to be parsed. **Returns:**: the list of elements corresponding to the provided string.

---


### getListFormat

```
public ListFormat<E> getListFormat()
```

:   Returns the underlying list format.

    :   **Returns:**: the underlying list format.

---


### getElementFormatter

```
public Formatter<E> getElementFormatter()
```

:   Returns the formatter for
    the elements of this list format.

    :   **Returns:**: the formatter for the elements of this list format.

---


### getElementParser

```
public Parser<E> getElementParser()
```

:   Returns the parser for
    the elements of this list format.

    :   **Returns:**: the parser for the elements of this list format.


---


|  |  |  |
| --- | --- | --- |
| **Overview** \| **Package** \| **Class** \| **Use** \| **Tree** \| **Deprecated** \| **Index** \| **Help** | |  |
| **PREV CLASS**   **NEXT CLASS** | **FRAMES**    **NO FRAMES**     **All Classes** |  |
| SUMMARY: NESTED \| FIELD \| CONSTR \| METHOD | DETAIL: FIELD \| CONSTR \| METHOD |  |


---
